# Microbiological Spectrum and Demographic Distribution of Pathogens Causing Suppurative Inflammation of the Cervical Lymph Nodes in Patients Over 18 Years of Age: An Eight-Year Retrospective Study

**DOI:** 10.7759/cureus.89951

**Published:** 2025-08-12

**Authors:** Yanko G Yankov, Lyuben L Stoev, Martina G Stoeva, Alexandar L Stoev, Iliana D Mechkarova, Diana D Nenova

**Affiliations:** 1 Clinic of Maxillofacial Surgery, University Hospital "St. Marina", Varna, BGR; 2 Department of General and Operative Surgery, Medical University "Prof. Dr. Paraskev Stoyanov", Varna, BGR; 3 Clinic of General and Clinical Pathology, University Hospital "St. Marina", Varna, BGR; 4 Department of General and Clinical Pathology, Forensic Medicine and Deontology, Medical University "Prof. Dr. Paraskev Stoyanov", Varna, BGR; 5 Faculty of Dentistry, Medical University "Prof. Dr. Paraskev Stoyanov", Varna, BGR; 6 Second Department of Internal Disease, Medical University "Prof. Dr. Paraskev Stoyanov", Varna, BGR; 7 Clinic of Nephrology and Dialysis, University Hospital "St. Marina", Varna, BGR

**Keywords:** abscess, antibacterial treatment, cervical lymphadenitis, cervical lymph nodes, etiological bacterial spectrum, head and neck pathology, maxillofacial surgery, neck infection, phlegmon, pus

## Abstract

Introduction

Purulent inflammatory diseases of the head and neck represent the most common pathology within the domain of oral and maxillofacial surgery that necessitates hospitalization and urgent surgical intervention. A considerable proportion of these cases involve inflammation of the cervical lymph nodes. If not treated promptly, such infections can lead to prolonged hospital stays, including admission to intensive care units, impose a significant burden on healthcare systems, and result in severe complications, including death.

Materials and methods

This original study is a retrospective analysis conducted over an eight-year period (2015-2022), involving 26 adult patients (14 men and 12 women) diagnosed with purulent inflammation of the cervical lymph nodes who underwent emergency incision and drainage. In all cases, biological specimens were collected intraoperatively for microbiological analysis. The obtained results were processed and analyzed in the present work.

Results

The mean patient age was 49 years (range 23-81), with an average of 43 years (range 23-72) in men and 55 years (range 29-81) in women. Microorganisms were isolated in 23 of 26 patients. The most frequently identified pathogen was *Staphylococcus aureus* (n = 15), followed by resident oral microflora (n = 4), *Klebsiella pneumoniae* (n = 2), *Enterobacter cloacae* (n = 1), and *Candida albicans* (n = 1). In three patients, no microorganisms were detected (sterile cultures).

Conclusions

Our study, although single-center, confirms that in cases of purulent cervical lymphadenitis within the scope of oral and maxillofacial surgery, empirical antimicrobial therapy should be selected to provide coverage for both Gram-positive and Gram-negative organisms. Clinicians should also be aware that, although uncommon, fungal pathogens may be encountered.

## Introduction

Inflammatory diseases of the head and neck are the most common conditions leading to emergency hospitalization of patients in maxillofacial surgery clinics, both in adults and in the pediatric population [1,2]. If not diagnosed and treated in a timely manner, these diseases are associated with serious, including fatal, complications such as cavernous sinus thrombosis, mediastinitis, and sepsis [1-3]. In a large number of patients, purulent-inflammatory diseases of the head and neck have a prolonged course, requiring a stay in an intensive care unit and daily medical care for both the postoperative wounds and the deteriorated general condition of the patients [4]. This drastically increases the cost of treatment and is associated with a significant burden on the medical staff involved in their care [2,3]. Understanding the demographic characteristics and the etiological microbial spectrum of these severe infections is crucial for their proper treatment, prognosis, and final outcome [1,5]. There are a number of studies on the etiological bacterial spectrum of purulent examinations of the head and neck, but most of them analyze the causative agents of extranodal inflammations, such as abscesses and phlegmons. In contrast, the present scientific work identifies and analyzes the etiological agents of purulent inflammation of the cervical lymph nodes themselves.

The aim of this study is to investigate the microbiological spectrum and demographic distribution of the pathogens responsible for suppurative inflammation of the cervical lymph nodes in hospitalized patients aged 18 and older in Northeastern Bulgaria. This includes identifying the most common causative microorganisms, analyzing patient demographics (e.g., age and sex distribution), and assessing the implications for empirical and targeted antimicrobial therapy.

## Materials and methods

The present study, conducted at the Clinic of Maxillofacial Surgery at University Hospital "St. Marina," Varna, Bulgaria, is retrospective in nature and covers an eight-year period (from January 1, 2015, to December 31, 2022). The medical records of a total of 6,763 patients (both hospitalized and outpatient) with head and neck diseases who were treated at the clinic during the study period were reviewed. From this group, patients were selected based on the following predefined criteria: 18 years of age or older, hospitalization, underwent surgical treatment, and diagnosed with cervical lymph node abscess. The diagnosis was based on the following three diagnostic criteria: a painful infiltrate with palpatory evidence of fluctuation in the neck region, identified during physical examination; purulent exudate in the cervical lymph nodes, confirmed by imaging studies (ultrasound, magnetic resonance imaging, or contrast-enhanced computed tomography); and presence of pus from the inflamed cervical lymph nodes observed during surgery (incision, lavage, and drainage).

All patients underwent microbiological testing at the University Microbiology Laboratory on intraoperatively collected material from the affected cervical lymph nodes, including cultivation on solid (blood agar, chocolate agar, MacConkey agar, Sabouraud agar, and Columbia agar) and liquid (soy-casein and thioglycolate broth) media with subsequent incubation under aerobic and anaerobic conditions. Species identification was done by manual biochemical tests and the BD Phoenix automated system (Becton Dickinson, MD, US). The Kirby-Bauer disc diffusion method and BD Phoenix automated system were used for antimicrobial susceptibility testing.

The exclusion criteria included individuals under 18 years of age, non-hospitalized patients, patients not meeting all three diagnostic criteria, and cases with incomplete medical documentation. A total of 26 patients, all hospitalized on an emergency basis, met the inclusion criteria and were included in the study. All patients underwent microbiological testing at the University Microbiology Laboratory using intraoperatively obtained material from the cervical lymph nodes.

## Results

**Figure 1:**
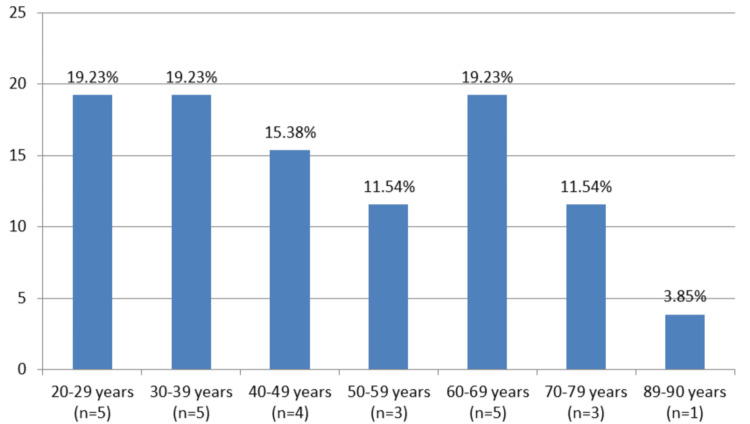
Age distribution of the 26 studied patients diagnosed with purulent cervical lymphadenitis The abscissa represents the individual age groups and the number of individuals in each of them (n), and the ordinate represents their percentage ratio (%).

During the period 2015-2022, a total of 26 patients with purulent cervical lymphadenitis who met the inclusion criteria outlined in the Materials and Methods section were studied. Men slightly predominated (n = 14, 53.8%). The mean and median age of the patients were 53.5 and 57 years, respectively (IQR: 67-37). The age distribution of the patients is presented in Figure 1.

Microbiological confirmation was achieved in 88.5% of the cases (23/26). In three patients, no microbial flora was detected in the intraoperatively obtained clinical material (11.5%; 3/26).

**Figure 2:**
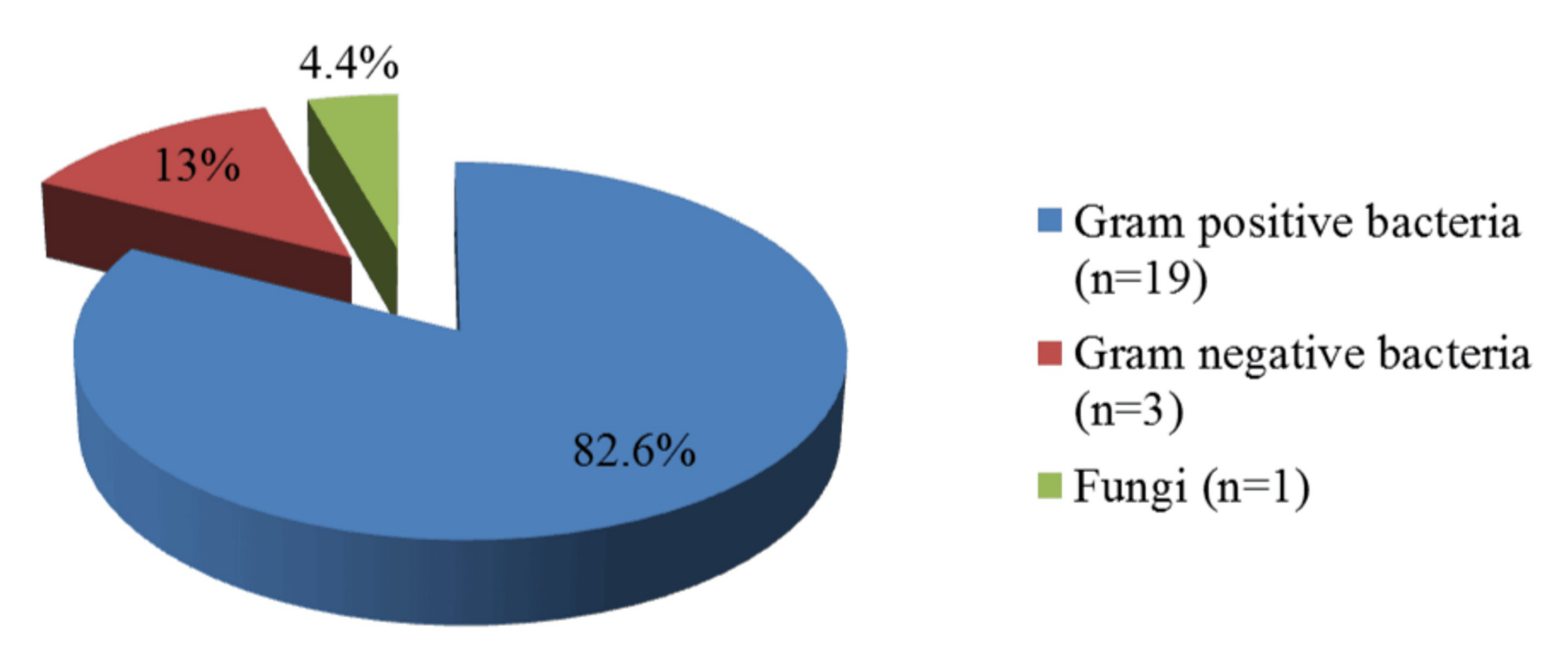
Spectrum of pathogens in microbiologically confirmed purulent cervical lymphadenitis in 23 hospitalized patients

Among the isolated microorganisms associated with purulent cervical lymphadenitis, Gram-positive bacteria accounted for 82.6% of the isolates (Figure 2). The ratio of Gram-positive to Gram-negative bacteria, based on the total number of bacterial isolates for the period 2015-2022, was 82.6% to 13%. Fungal organisms, represented by yeast-like fungi of the genus *Candida* (*Candida albicans*), constituted 4.4% of all microbial isolates.

**Figure 3:**
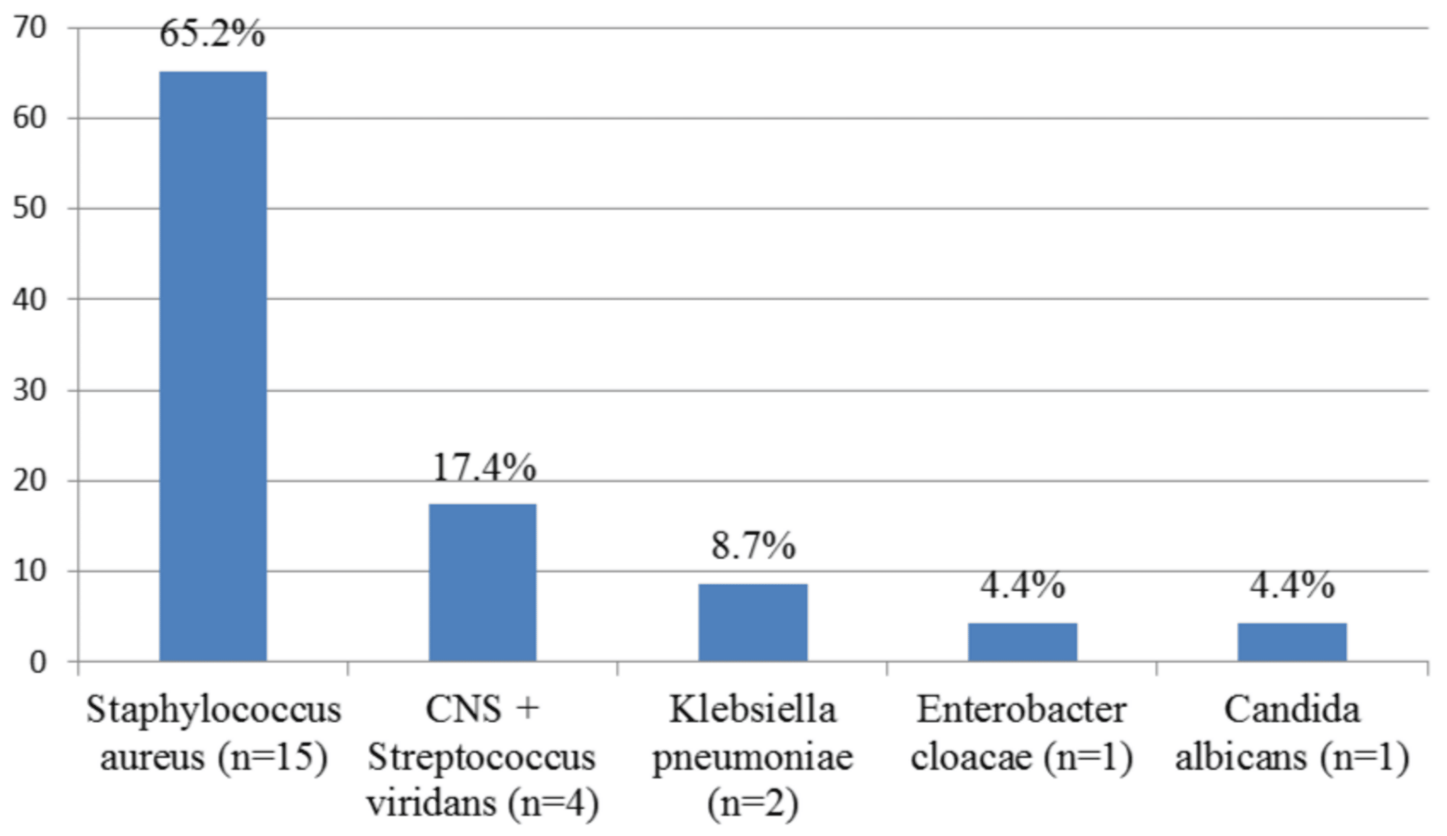
Detailed etiological structure of purulent cervical lymphadenitis in 23 hospitalized patients with microbiologically confirmed pathogens The abscissa represents the isolated pathogens and their number (n), and the ordinate represents their percentage ratio (%).

The etiological structure of purulent cervical lymphadenitis by microbial species is presented in Figure 3.

*Staphylococcus aureus* was identified in 65.2% of all microbiologically confirmed cases (15/23). In four patients (17.4%; 4/23), an association was identified between the etiological agents and isolates of Gram-positive bacterial species that are part of the resident oral microflora - coagulase-negative *Staphylococcus* (CNS) and *Streptococcus viridans*.

## Discussion

Lymph node inflammation, if not controlled in a timely manner, progresses through three consecutive stages [2]. In the first stage, serous inflammation, the infection is confined within the lymph node itself (limited by its capsule), and the lymph node retains its shape, remains mobile, and is distinguishable from surrounding tissues [2,6]. It is variably painful, and patients usually do not present with systemic symptoms [2,7].

If treatment is not initiated, and in cases of weakened natural resistance and/or immunity of the host organism or high pathogen virulence, the infection advances to the second stage of inflammation. This stage is purulent in nature, with the formation of a pus focus that remains confined within the affected lymph node. The surrounding soft tissues become edematous and painful upon palpation, and the lymph node itself becomes difficult to differentiate. Systemic symptoms may appear at this stage - most commonly low-grade or high-grade fever [2,6,7].

In the third stage of the disease, the capsule of the lymph node is lysed, and the purulent secretion spreads into the adjacent soft tissue spaces. This stage, known as lymphadenophlegmon, presents clinically with systemic intoxication and fever - symptoms characteristic of abscesses and phlegmons in soft tissue compartments. The affected anatomical region is intensely painful, and fluctuation can be detected on palpation due to the presence of a purulent collection [2,8].

The serous stage of lymph node inflammation requires only symptomatic and antibacterial therapy, whereas the presence of pus - characteristic of the more advanced stages of infection - necessitates surgical intervention for the evacuation of the purulent material, which is critically important [9,10]. The appropriate choice of antibiotic therapy, particularly empirical treatment in emergency settings, directly depends on knowledge of the spectrum of etiological agents responsible for lymphadenitis - one of the main objectives of this study [2,11,12].

This study demonstrates that in the examined group of patients, selected based on predefined criteria over the entire eight-year period (2015-2022), Gram-positive bacteria predominate as the causative agents of purulent lymphadenitis in hospitalized patients over 18 years of age, with *S. aureus* being the leading pathogen. This bacterial species shows clear dominance not only among the Gram-positive isolates but also across all confirmed etiological agents in the study.

*S. aureus* is one of the most clinically significant microorganisms, responsible for both community-acquired infections and healthcare-associated infections. This microorganism colonizes various parts of the body such as the skin, nasal passages (its primary ecological niche), and the axillary and inguinal regions. Approximately 25%-30% of healthy individuals in the human population are colonized by *S. aureus*, with around 15% of them being persistent carriers [13]. *S. aureus* is associated with three main types of disease: toxin-mediated diseases, suppurative infections, and infections of various anatomical localizations (including lymphadenitis and lymphangitis), as well as secondary invasive infections such as bacteremia, endocarditis, sepsis, and septicopyemia [14].

In the human body, staphylococci spread via the lymphatic pathway from the portal of entry of the infection to the local lymph nodes, where they cause purulent inflammation. The pathogenicity of *S. aureus* is mediated by a variety of virulence and pathogenicity factors, including the capsule, slime, clumping factor, protein A, exotoxins, adhesins, enzymes, and mechanisms of antibiotic resistance [15,16].

The normal microflora of the oral mucosa includes staphylococci other than *S. aureus* (CNS) and streptococci from the *S. viridans* group. This may explain the easy access of these microorganisms to the lymph nodes located adjacent to these anatomical regions (regional lymph nodes) [2,16]. The present study identifies these Gram-positive bacteria, found in association (CNS + *S. viridans*), as etiological agents in 17.4% of the cases, ranking second in frequency after *S. aureus*.

It should be noted that the normal oral microflora also includes obligate anaerobic microbial species such as *Fusobacterium*, *Actinomyces*, *Porphyromonas*, *Prevotella*, *Eubacterium*, and *Leptotrichia* [17]. This is the reason why some infections in this anatomical region - particularly abscesses, including those affecting lymph nodes - may have a mixed aerobic-anaerobic etiology or be associated solely with anaerobic bacteria.

Obligate anaerobic bacteria are extremely sensitive to oxygen, which is highly toxic to them even in small quantities, including during brief exposure (e.g., aeration of the clinical sample during surgery or improper transport to the microbiology laboratory). This may explain why, despite convincing signs of inflammation, clinical specimens may remain sterile and infections microbiologically unconfirmed. Successful isolation and identification of obligate anaerobes is a more complex diagnostic process that requires compliance with several conditions related to the type of clinical material (the most appropriate being aspirates or tissue fragments), as well as proper storage, transport, and the creation of suitable incubation and culture conditions.

In the present study, no microbial flora was isolated from the intraoperatively obtained biological materials of three patients. Possible reasons for this include anaerobic etiology of the infection, failure to meet sampling and transport requirements, or testing of biological material composed only of pus, which essentially consists of leukocytes that have phagocytosed and destroyed microbial cells and, as a result, is often sterile. Although at a significantly lower relative proportion, the present study confirms the presence of Gram-negative facultative anaerobic bacteria - specifically *Klebsiella pneumoniae* and *Enterobacter cloacae*, members of the order Enterobacterales - within the etiological spectrum of purulent cervical lymphadenitis.

These two bacterial species are part of the normal gastrointestinal microflora of humans and animals, but they are also found in environmental sources such as soil and water [18]. They are not part of the normal microflora of the upper respiratory tract or oral cavity; however, their presence in these anatomical regions - a condition known as colonization - is typical in older patients, individuals who have undergone prolonged antibiotic therapy, or those who are immunocompromised. Such colonization may serve as a risk factor for subsequent infection [19]. At present, *K. pneumoniae* and *E. cloacae* species are recognized as classic opportunistic pathogens, responsible for a wide range of infections - including lymphadenitis - often nosocomial in nature and primarily affecting immunosuppressed individuals. These infections typically arise following the invasion of sterile tissues when the integrity of mechanical barriers (such as skin or mucosa) is compromised. This mechanism likely explains how these bacteria reach the lymph nodes and trigger subsequent inflammation [19,20].

Yeast-like fungi of the genus *Candida*, with *C. albicans* being the most common representative, are part of the normal gastrointestinal microflora in approximately 40% of people. These microorganisms are typically present in very low microbial counts and do not cause disease under normal conditions [21].

Overgrowth of *Candida* in the gastrointestinal tract represents a state of dysbiosis, which may be linked to various factors, including the use of broad-spectrum antimicrobial agents [22]. Candidiasis as a clinical condition is more frequently observed in immunocompromised patients (e.g., individuals with HIV, transplant recipients, and cancer patients), while oral candidiasis is more commonly seen in elderly patients, denture wearers, or those with foreign bodies (e.g., tongue piercings) [23,24].

In our study, *C. albicans* was identified as an etiological agent in only one patient. Detection of fungal pathogens as the cause of lymphadenitis necessitates reassessment and adjustment of antibiotic therapy, which is typically initiated empirically in patients with purulent infections [2,25,26]. The ability to tailor therapy based on etiological diagnosis and the resulting improvement in patient prognosis and clinical outcomes highlight the crucial role of microbiological testing as a key element in the management of purulent lymphadenitis.

Limitations

The present study has several limitations. It is single-center and retrospective in nature, includes a relatively small number of patients from a single geographic location, and does not involve individuals under the age of 18. In addition, the study sample is small and assesses only cervical lymph nodes.

## Conclusions

The present study identifies Gram-positive bacterial species, either alone or in association, as the leading causative agents of purulent infections of the cervical lymph nodes in patients aged 18 years and older. *S. aureus* is confirmed as the most significant pus-producing Gram-positive organism, responsible for over 50% of the cases. Gram-negative bacteria, represented by *K. pneumoniae* and *E. cloacae*, as well as yeast-like fungi of the genus *Candida*, were detected at a very low relative proportion. The study did not identify any cases of purulent cervical lymphadenitis associated with anaerobic bacteria or mixed aerobic-anaerobic microflora. With this, our study confirms the already existing data on the topic about the etiological spectrum of acute purulent cervical lymphadenitis.

Despite the dominant etiological role of Gram-positive bacteria, empirical antimicrobial chemotherapy in patients with purulent cervical lymph node infections should target a broader spectrum of microorganisms, including anaerobic bacteria, and always consider the possibility of fungal infection. Performing microbiological testing as a mandatory part of the diagnostic and therapeutic plan in such patients enables the application of targeted etiological therapy and timely escalation or de-escalation of antibiotic treatment.
